# A Case of Lupus Myopericarditis in a Young African American Male

**DOI:** 10.7759/cureus.79690

**Published:** 2025-02-26

**Authors:** Chirag Lodha, Mohamed A Abutineh, Nazar Sharak

**Affiliations:** 1 Internal Medicine, Edward Via College of Osteopathic Medicine, Spartanburg, USA; 2 Medicine, Edward Via College of Osteopathic Medicine, Spartanburg, USA; 3 Cardiovascular Medicine, Cleveland Clinic Florida, Vero Beach, USA

**Keywords:** acute myopericarditis, autoimmune flare up, autoimmune pericarditis, lupus flare, pleuritic chest pain

## Abstract

A 28-year-old African American male presented to the emergency department with a chief complaint of chest pain. He stated that the pain began five days prior to arrival and had not improved despite taking ibuprofen and self-medicating with marijuana. His chest pain was constant and localized to the center of his chest, rating it as a 7/10 in terms of severity. His past medical history included a diagnosis of systemic lupus erythematosus (SLE) three months ago, complicated by pericarditis, for which he had been taking colchicine and prednisone, as well as hydroxychloroquine for maintenance therapy. He stated that he adhered to his treatment regimen without missing a single dosage. Upon cardiac auscultation, he was found to have a pericardial friction rub. His ECG showed signs of PR-segment depression in all leads, ST-elevation in leads V2-V4, as well as sinus tachycardia, raising some concern for myocardial ischemia. His echocardiogram showed no signs of pericardial effusion or left ventricular dysfunction and was otherwise unremarkable. Because of his positional chest pain, diffuse ST-elevation, and elevated troponin, the patient was diagnosed with myopericarditis, thought to be secondary to SLE exacerbation due to no recent illnesses. He was then placed on low-dose prednisone. After four days, his symptoms resolved, and he was discharged with a regimen of 60 mg prednisone daily and 0.5 mg colchicine daily for his pericarditis as well as 10 mg oxycodone for his pain every eight hours. His prednisone was to be tapered with a rheumatologist outpatient, but there was no follow-up at the time of writing this study.

## Introduction

Systemic lupus erythematosus (SLE) is an autoimmune condition that can affect a diverse variety of organs, with symptoms ranging anywhere from mild joint pain to kidney failure [1]. It does so by creating antibody complexes that deposit in blood vessels and tissues, causing organ damage and malfunction. Although the cause of SLE is unknown, there is strong evidence for a multifactorial model, implicating over 100 susceptibility genetic loci in the pathogenesis of SLE [2]. It initially manifests with nonspecific symptoms, such as fatigue, myalgias, and arthralgias, seen in over 50% of SLE patients. Complications of SLE can be due to the inherent nature of inflammation of autoimmune diseases as well as antibody deposition. However, it is unknown why SLE targets some organ systems over others [1].

In one meta-analysis, the prevalence of lupus was 73 per 100,000 [2]. However, African American males represent only 0.7% of these SLE cases in the United States, making this demographic an under represented population in SLE literature [3]. Due to the rarity of SLE diagnoses in African American males, a high level of clinical suspicion must be held to make this diagnosis. Most African American men are diagnosed with lupus between the ages of 45 and 64, making it a relatively uncommon diagnosis for younger African American patients. African American males tend to have greater cardiovascular disease and overall disease severity than African American women [2]. Cardiac complications of SLE include pericarditis, accelerated coronary disease, and thromboembolic disease [1]. These complications are less common (20% of SLE patients) than more well-known organ involvement such as the kidney (50% of SLE patients). However, they account for a significant proportion of morbidity and mortality due to the inflammatory nature of autoimmune diseases and accelerated atherosclerosis. Out of the numerous cardiac manifestations of SLE, the most common is pericarditis. Pericarditis is defined as inflammation of the outer lining of the heart, also known as the pericardium. This is most often due to a viral infection [4]. However, this is also seen secondary to other systemic diseases, such as autoimmune diseases causing immune complex deposition or inflammation of the pericardium.

Patients classically present with chest pain that improves with sitting up and forwards, with ECG findings showing widespread ST-segment elevation as well as PR-segment depression [4-5]. Upon cardiac auscultation, a pericardial friction rub is heard over the left sternal border. This is most often described as a scratching sound, heard best with the diaphragm of the stethoscope. Additionally, additional consideration must be made for an ST-segment elevation myocardial infarction, as these patients often develop significant coronary artery disease at an earlier age. An important consideration is that in patients with an underlying rheumatologic disorder, chest pain may be minimal and, in some cases, even absent, so emphasis must be placed on ECG as well as physical examination for the diagnosis of acute pericarditis in this patient population [5].

However, a minority of SLE pericarditis patients will present with chest pain during their first lupus exacerbation. Therefore, specifically, in the case of SLE, chest pain may not be a reliable physical exam finding to raise the suspicion of pericarditis, especially as over 50% of SLE patients with pericarditis are often asymptomatic. Therefore, there must be a high level of suspicion in SLE patients with ECG changes regardless of the presence of chest pain. Atypical symptoms may include fatigue or dyspnea in these patients and should therefore raise suspicion of pericarditis. In cases of severe pericardial inflammation, pericarditis can evolve into myopericarditis, characterized by inflammation and damage of the muscular layer of the heart as well as the pericardium [5]. Patients with characteristic findings of pericarditis with elevated cardiac troponin biomarkers indicating myocardial damage are diagnosed with myopericarditis [6]. Approximately 3-6% of SLE patients with pericarditis progress to myopericarditis [4]. Some risk factors for the development of SLE pericarditis include hemolytic anemia, proteinuria, and anti-Smith antibodies. Antibody deposition can lead to direct immunofluorescence showing granular immune complex deposition in the pericardium on histology [7].

In the case of viral or idiopathic acute pericarditis, the first-line treatment is NSAIDs and colchicine. Therapy duration is determined in one of two ways: one is by basing treatment duration on the resolution of symptoms, while another is based on both the resolution of symptoms, as well as normalization of C- reactive protein (CRP) titers [8]. Although elevation of CRP is often seen in the context of any inflammation or infection, it can be used as a valuable tool to gauge the severity of an autoimmune disease exacerbation as well as patient response to treatment. A study done with 200 cases of viral or idiopathic acute pericarditis showed that high-sensitivity CRP was an independent risk factor for the recurrence of pericarditis and should therefore be an additional consideration during initial therapy during a first episode of pericarditis [9].

However, studies have not shown a significant difference in either of these approaches when deciding therapy duration [8]. Between 15-30% of patients will have a recurrence of pericarditis [7]. Due to the inherent nature of autoimmune diseases, these patients are potentially at a higher risk for the development of recurrent pericarditis secondary to systemic inflammation and antibody deposition. In cases of systemic autoimmune diseases, glucocorticoids are the first-line treatment, as these patients often fail treatment with NSAIDs and colchicine. Glucocorticoids can also be used in refractory cases of idiopathic and viral pericarditis. Prednisone is usually the glucocorticoid of choice, as it has shown efficacy in down-regulating the immune system in exacerbations of autoimmune diseases. In cases refractory to glucocorticoids, other immunosuppressive medications, such as mycophenolate, can be used [8].

## Case presentation

A 28-year-old African American male presented to the ED complaining of chest pain. He states that he had been dealing with it for the past week and was self-medicating with marijuana as well as ibuprofen to help with the pain. He described the pain as bearable but rated it as 5/10 in the center of his chest. However, the night before his admission, he noticed that despite taking ibuprofen, the pain did not improve as it had in the past. As he was worried his chest pain might be something serious, he came to the emergency department via ambulance.

Upon arrival at the emergency department, he did not appear to be in acute distress but was mildly uncomfortable, which he attributed to central chest pain. He described the pain as 7/10, non-radiating, and worsening when lying on either his left or right side. There was no specific time of day when the pain was worse. He could only sleep propped up on a pillow in bed or in his recliner. He denied any recent illnesses and noted that the chest pain prevented him from taking deep breaths.

When asked about shortness of breath, he acknowledged experiencing it. Further review of systems revealed that he also reported lower leg pain in both shins. He described the pain as a deep, sharp sensation, well localized to the anterior aspect of his tibia, but it did not affect his ability to bear weight on either leg. There was no trauma that he could recall, and he stated that this had happened once before three months ago, when he also went to the ED with similar bone and chest pain, although not as severe. This was when he was diagnosed with SLE, for which he had been taking colchicine and prednisone for his pericarditis, up until two weeks ago when he finished his treatment course. He had also been taking hydroxychloroquine as maintenance therapy for SLE. His SLE was well managed, and he had no exacerbations with his treatment regimen since he was diagnosed.

**Figure 1:**
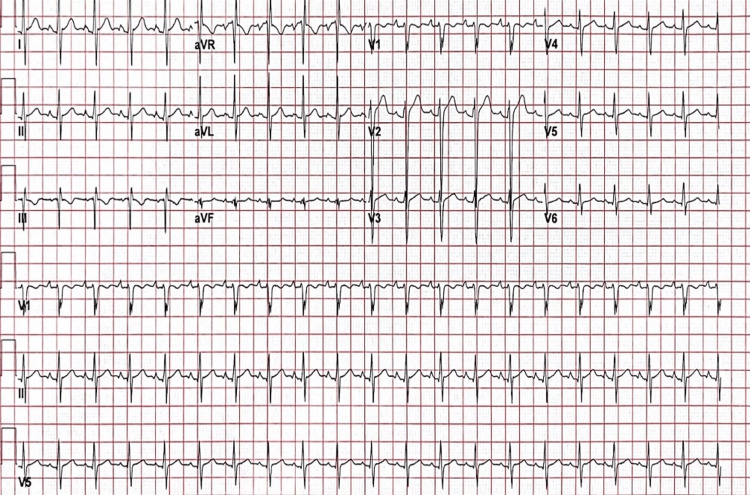
Patient ECG showing ST-elevation with PR depression.

During his initial episode, he did not experience chest pain but presented with bone and joint pain. Aside from this, he had no significant medical, surgical, or family history of relevant conditions. His vital signs were largely unremarkable, except for tachycardia with a heart rate of 105 beats per minute, and he remained afebrile. On physical examination, he had no reproducible chest pain with palpation. When asked to breathe deeply, he was unable to inhale maximally due to his pain. Cardiac auscultation showed a fast heart rate with a regular rhythm. A pleuritic friction rub was also noted. His arms showed spotty areas of depigmentation on the flexor aspects. Due to his chest pain, an ECG was ordered as well as serial high-sensitivity troponin, showing ST elevation in leads V2 through V6 as well as PR-depression throughout the majority of leads (Figure 1).

His troponin levels were markedly elevated based on hospital criteria. However, due to his positional discomfort and shortness of breath, his presentation was inconsistent with acute coronary syndrome. He was monitored while undergoing further evaluation. His CMP and X-ray in the ED were unremarkable, having been ordered to assess for pulmonary embolism, aortic dissection, or potential organ damage.

However, his CBC showed microcytic anemia with high ferritin and low TIBC, suggestive of anemia related to chronic disease. This supported the autoimmune reason for his chest pain, as it indicated underlying inflammation preventing the formation of red blood cells, ruling out any concern for an active bleed. Additionally, his CRP was elevated, indicating underlying inflammation. CRP can be elevated in numerous conditions, but a baseline for patients with a well-controlled autoimmune disease can help determine when an exacerbation occurs, as CRP will become markedly elevated during a flare-up. Subsequently, serum antibodies were drawn. His antibody panel came back positive for several antibodies, notably anti-Sjögren’s syndrome-related antigen A antibody (anti-SSA) and anti-ribonucleotide protein (anti-RNP) as shown in Table 1.

**Table 1:** Pertinent patient lab values. CRP: c-reactive protein; Anti-RNP: anti-ribonucleotide protein. ANA: antinuclear antibody; Anti-SSA: anti-Sjogren's syndrome-related-antigen-A antibody.

Parameters	Patient value	Reference range
High-sensitivity troponin	1100 ng/L	<52 ng/L
CRP	13.3 mg/dL	<0.9 mg/dL
Anti-RNP antibody	+	N/A
ANA antibody	+	N/A
Chromatin antibody	+	N/A
Anti-SSA antibody	+	N/A
Prothrombin time	20.5 seconds	11.6-14.4 seconds
Hemoglobin	6.8 g/dL	10.0-18.5 g/dL

In the context of the patient’s diagnosis, these antibodies are seen in SLE, but also in Sjogren’s (anti-SSA) and mixed connective tissue diseases (anti-RNP), indicating that the patient may have begun to develop other autoimmune conditions that required further rheumatologic testing. However, the decision to follow outpatient for further workup was made by the rheumatology team after discharge. His echocardiogram was unremarkable, with no signs of pericardial effusion, left ventricular dysfunction, or any cardiac hypokinesis, further ruling out an ACS. Due to his diffuse ST elevations on ECG, elevated troponin levels, and the presence of a pleuritic friction rub on physical examination, he was diagnosed with acute myopericarditis, likely secondary to a lupus exacerbation, given his positive antibody titers and history of autoimmune disease.

He was prescribed oxycodone/acetaminophen (Percocet, Endo Pharmaceuticals, Inc.) for pain management, as NSAIDs and tramadol failed to control his pain in the emergency department. He was also given morphine in case of breakthrough pain. Although there was a risk for opioid dependence, the severity of pain combined with the short course of opioids was deemed to be of more benefit and was therefore prescribed. The first line treatment for viral or idiopathic pericarditis is either NSAIDs or colchicine and was preferred by cardiology. 

However, rheumatology was also consulted due to the patient’s lupus exacerbation as evidenced by elevated CRP, and believed that low-dose prednisone would be a better option to resolve the pericarditis, as it was secondary to a systemic autoimmune process. Ultimately, the patient was placed on low-dose prednisone at a dosage of 60 mg/daily with his pericarditis and bone pain resolving by his fourth day in the hospital. The patient remained pain-free by this time.

He was discharged with oxycodone as needed for pain, colchicine for pericarditis management, and hydroxychloroquine for SLE, along with instructions to follow up with a rheumatologist to discuss a prednisone taper. He was also educated on signs of opioid dependence and overdose as well as alternative pain management with NSAIDs if his pain was not as severe. He was told to remain vigilant for signs of an additional exacerbation, such as chest pain, fatigue, myalgias, bone pain, or shortness of breath. He was instructed to see cardiology and nephrology within one week of discharge but had not yet done so at the time of writing.

## Discussion

While interviewing this patient upon his initial presentation, his primary concerns were his chest pain as well as his bone pain over his anterior shins, as both prevented him from sleeping more than three to four hours at night since the start of his symptoms. Therefore, pain management was of utmost importance while conducting further tests and drawing additional labs on this patient. For this reason, he was placed on NSAIDs and tramadol in the ED. However, his pain did not improve with this regimen, and he was then placed on percocet as well as morphine for breakthrough pain. The patient’s pain persisted despite NSAIDs and tramadol, likely due to the severity of inflammation associated with lupus myopericarditis. No adverse effects were noted, but inadequate pain control necessitated opioid use. Opioids carry risks such as dependence, respiratory depression, and gastrointestinal side effects. Long-term opioid therapy in patients with SLE is especially concerning, given their underlying increased risk for osteoporosis and cardiovascular disease [8]. However, due to the patient’s significant pain and failure of first-line analgesics, oxycodone was prescribed under close monitoring for a limited duration.

As his ECG showed signs of diffuse ST-elevation in leads V2-V4, an ST-elevation myocardial infarction (STEMI) was ruled out despite his high troponin as his chest pain was positional. Elevated troponin and ST-elevations are not exclusive to STEMI, especially in the absence of other specific clinical features like radiation of pain or a family history of coronary artery disease. Diffuse ST-elevation is one of the diagnostic criteria for myopericarditis. Myopericarditis is also distinguished by positional chest pain, systemic inflammatory markers, and an absence of coronary ischemia [5]. STEMI must be ruled out due to its life-threatening implications and the requirement for urgent intervention. In a study of 816 patients with SLE, the leading cause of death after being followed for over a decade was cardiovascular disease, accounting for one-third of patient deaths [10]. Due to the advanced coronary artery disease present in SLE patients, ST-elevation myocardial infarctions must be considered, as they can mimic the presentation of myopericarditis due to ST-elevation found on ECG as well as elevated cardiac troponin markers, and must therefore be ruled out before other diagnoses [6].

SLE contributes to cardiovascular disease through chronic inflammation, endothelial dysfunction, and accelerated atherosclerosis. Endothelial dysfunction is known to be a key event in atherogenesis, and chronically elevated levels of inflammatory markers may contribute to endothelial damage. An acute phase protein used to measure inflammation in autoimmune processes, CRP, also plays an important role in atherogenesis by way of involvement in plaque growth. Autoantibody-mediated damage and prothrombotic states further exacerbate risk, making cardiovascular monitoring crucial in these patients.

Recurrent pericarditis is also a concern in patients with SLE. Recurrent pericarditis is defined as an episode of acute pericarditis that occurs at least 4 to 6 weeks after the resolution of a prior episode. Recurrent episodes can occur months or even years after the initial onset. Since our patient’s last episode of pericarditis occurred three months ago, this qualifies as recurrent pericarditis. Patients with autoimmune diseases make up the majority of recurrent pericarditis cases, as both the underlying condition and prednisone therapy serve as risk factors. However, it is unknown how many patients with SLE suffer from recurrent pericarditis as it can often be asymptomatic. The first-line treatment for recurrent pericarditis is the same as acute pericarditis: NSAIDs in combination with colchicine [11]. Glucocorticoids were selected because they are specifically indicated for recurrent pericarditis associated with systemic inflammatory diseases, even though they are considered a second-line option for recurrent pericarditis in the absence of an underlying autoimmune condition [11].

There were numerous risks that had to be addressed before starting glucocorticoid therapy. Patients with SLE often have an underlying vitamin D deficiency, often due to renal involvement in the disease. As a result, they are at increased risk of developing osteopenia and, potentially, osteoporosis. By starting glucocorticoid therapy, the risk of osteoporosis in these patients increases, as glucocorticoids have been known to lead to decreases in bone density [12]. The patient had been on low-dose prednisone for the past three months and would likely be placed on it again for his myopericarditis. He was therefore at a higher risk for osteoporosis and potential fractures. The risk of fractures in patients with SLE is approximately five times that of the general population [12]. Ultimately, his risk of fracture was outweighed by that of his myopericarditis, and he was therefore placed on glucocorticoid therapy for his myopericarditis refractory to NSAIDs.

Glucocorticoids were preferred due to the autoimmune nature of the patient’s pericarditis. While NSAIDs and colchicine are first-line for viral/idiopathic pericarditis, glucocorticoids are indicated in autoimmune-related cases due to their superior efficacy in suppressing inflammation. Chronic glucocorticoid use increases the risk of osteoporosis, immunosuppression, and metabolic disturbances. In this patient, short-term use was justified due to the severity of inflammation, but bone health monitoring is essential. Patients on long-term glucocorticoids should receive calcium and vitamin D supplementation and undergo bone density scans as indicated. The urgency of controlling inflammation outweighed immediate concerns for osteoporosis in this case.

Another way to prevent osteoporosis and fractures in patients is to encourage them to exercise, as light weight-bearing exercise has been shown to increase bone density [12]. However, in patients with active pericarditis, strenuous physical activity must be avoided until patients are asymptomatic, as this can exacerbate symptoms [12]. As previously stated, most SLE patients are asymptomatic when they have an episode of pericarditis. Therefore, it can be difficult to accurately gauge activity restrictions in this patient population and providers must take this into consideration before discharge. Additionally, it is important to start at a moderate dosage of glucocorticoids and slowly taper the dosage, replacing it with an NSAID or aspirin towards the end of therapy duration and adding colchicine as additional therapy. Low-dose corticosteroids have been shown to have superior outcomes than higher doses, as they lead to lower rates of recurrent pericarditis [8].

This patient’s case was also unique in his presentation of bone pain. The most common musculoskeletal complication that patients with SLE face is arthralgias, seen in about 95% of patients. These are most often migratory and symmetric [12]. However, bone pain is rare, and while it could indicate osteonecrosis or avascular necrosis in this patient due to his SLE and glucocorticoid usage, this usually happens at the femoral head or tibial plateau, not the lower anterior half of the patient’s tibia [12]. Additionally, these conditions would present with an inability to bear weight, which the patient tolerated well. Ultimately, there was no clear consensus as to what caused the patient’s anterior tibial pain. However, it resolved within a couple of days once the patient was placed on prednisone therapy. Although this patient presented in a very typical way for SLE patients with pericarditis, his age, gender, and race made his case a rare one. The evolution of his pericarditis into myopericarditis highlighted a very uncommon phenomenon seen in literature, further compounded by his atypical bone pain.

While lupus arthritis is common, bone pain is rare and typically linked to osteonecrosis or avascular necrosis. This patient’s pain was atypical, localized to the anterior tibia, and resolved with glucocorticoid therapy. During a literature search, there was not more than a handful of research articles discussing African American males with SLE and how outcomes differed between different races. However, these patients were between 45 and 64 years old [2].

SLE is more prevalent in African American females, and affected males tend to have more severe disease. Limited literature exists on young African American males with lupus myopericarditis, underscoring the need for further study. African American males with SLE may experience more severe disease manifestations, including cardiovascular and renal complications [2]. There was no case study or case series on young African American males suffering from SLE in the context of myopericarditis. This underscored the existing gap in literature and its need to be addressed. More research is needed to understand racial and gender disparities in SLE prognosis and treatment response.

## Conclusions

There are numerous possible manifestations of SLE during an exacerbation. Myopericarditis secondary to an SLE exacerbation is a rare complication of pericarditis. Due to its similar presentation to ACS, it can be frequently overlooked, especially since the SLE patient demographic has a high incidence of coronary disease that accounts for a significant amount of mortality. It presents with chest pain, elevated troponin markers, and ST-elevation on ECG. The treatment of pericarditis in SLE is complex, as there are many nuances in the treatment course as well as side effects. In the case of a viral etiology, NSAIDs and colchicine are the first line of treatment, while in autoimmune conditions, glucocorticoids are the first line. Chronic steroid treatment can lead to osteoporosis, accelerated coronary disease, as well as numerous previously discussed side effects contributing to significant patient morbidity. Alternative differential diagnoses include acute coronary syndrome, so providers should have a high degree of clinical suspicion in this patient population. This study aims to bring awareness to a clinically underappreciated population in current literature, as African American males are rarely diagnosed with SLE, especially those in their twenties, and when they are, their prognosis is far worse than any other patient demographic. 
